# Dynamic network measures reveal the impact of cattle markets and alpine summering on the risk of epidemic outbreaks in the Swiss cattle population

**DOI:** 10.1186/s12917-018-1406-3

**Published:** 2018-03-13

**Authors:** Beatriz Vidondo, Bernhard Voelkl

**Affiliations:** Veterinary Public Health Institute, University of Bern, Schwarzenburgstrasse 155, CH-3097 Liebefeld, Switzerland

**Keywords:** Cattle networks, Alpine pastures, Markets, Surveillance, Contact chain

## Abstract

**Background:**

Livestock herds are interconnected with each other via an intricate network of transports of animals which represents a potential substrate for the spread of epidemic diseases. We analysed four years (2012–2015) of daily bovine transports to assess the risk of disease transmission and identify times and locations where monitoring would be most effective. Specifically, we investigated how the seasonal dynamics of transport networks, driven by the alpine summering and traditional cattle markets, affect the risk of epidemic outbreaks.

**Results:**

We found strong and consistent seasonal variation in several structural network measures as well as in measures for outbreak risk. Analysis of the consequences of excluding markets, dealers and alpine pastures from the network shows that markets contribute much more to the overall outbreak risk than alpine summering. Static descriptors of monthly transport networks were poor predictors of outbreak risk emanating from individual holdings; a dynamic measure, which takes the temporal structure of the network into account, gave better risk estimates. A stochastic simulation suggests that targeted surveillance based on this dynamic network allows a higher detection rate and smaller outbreak size at detection than compared to other sampling schemes.

**Conclusions:**

Dynamic measures based on time-stamped data—the outgoing contact chain—can give better risk estimates and could help to improve surveillance schemes. Using this measure we find evidence that even in a country with intense summering practice, markets continue being the prime risk factor for the spread of contagious diseases.

## Background

Many livestock diseases can spread through direct contact between animals. Movement of animals from one herd to another can, therefore, lead to the spreading of highly infectious diseases [1–5]. The transport network of individuals from one holding to another is an important factor determining the spread of infectious diseases like, bovine tuberculosis, bovine viral diarrhoea, foot-and-mouth disease, and bovine coronavirus, amongst others [3, 6, 7]. Consequently, national registers for livestock transports have been implemented in many countries and the incorporation of network information in epidemic models and surveillance schemes has become an active area of research [8–13]. In these models the network is characterized as a graph, where holdings are represented by nodes and transports between holdings are edges. This allows for the calculation of quantitative descriptors of the connectivity (see Table 1 for definitions) and sub-structuring of the network as well as the positions of individual holdings in that network [14]. Dynamic approaches take into account the time sequence of the animal movements and allow more realistic models of transmission processes [15–18]. For example, Nörenmark et al. [19] and Frössling et al. [20] investigated to what extent a dynamic network measure—the ingoing contact chain—could be a useful measure when setting up strategies for disease control and for risk based surveillance.

**Table 1:** Definition and sources of the network analysis terms used in this study

Type / Name	Definition	Source
Holding centrality metrics		
In-degree	Number of individual sources providing animals to a specific holding	[14]
Out-degree	Number of individual recipients obtaining animals from a specific holding	[14]
Betweenness	The frequency a livestock operation is in the shortest path between pairs of holdings in the network	[47]
Closeness	The inverse of the sum of the shortest geodesic distances from a source holding to all other reachable holdings in the network	[48]
Static network measures of connectivity		
Geodesic	The shortest path length between two holdings	[49]
Path length	A path between farm A and farm C is the number of steps required to travel from A to C. In a path, holdings and animal transports cannot be repeated between a source and a destination. Related terms are geodesic and average path length	[49]
Average path length (APL)	The shortest path, or geodesic, among two holdings averaged over all pairs of holdings in the network	[50]
Density	Sum of the number of all links divided by the number of possible links in the network	[14]
Clustering coefficient (CC)	If a neighbour is defined as a the holding in direct contact with the holding of interest, the clustering coefficient represents the proportion of one’s neighbours who are also neighbours of one another	[50]
Components	Maximally connected subregions of a network in which all pairs of holdings are directly or indirectly linked	[46]
Giant strongly connected component (GSCC)	The largest component in the network in which all pairs of holdings are linked via directed paths	[43, 46]
Giant weakly connected component (GWCC)	The largest component in the network in which all pairs of holdings are linked regardless of the direction of the link	[43, 46]
Assortativity	Correlation between the degrees of linked premises	[51]

In cattle networks, markets have been identified as major contributors to network connectivity and thus, potential disease spread [21, 22]. Their exclusion has been shown to substantially reduce potential outbreak size [22, 23]. Likewise, Webb and colleagues [15] found that agricultural shows increase the potential for epidemic outbreaks in British sheep. Both markets and shows can be described as gathering events, where animals from many holdings are brought to the same location for a short period of time and are again distributed to many holdings, afterwards. Gathering events of both human and animals can drastically increase the risk of acquiring and transmitting disease [24, 25]. In network terms gathering events can be described as ‘hubs’—nodes with high in- and out-degree, connecting a high number of holdings. The existence of hubs in a network can lead to a highly skewed or heavy tailed distribution, which has important implication for the spread of diseases [26, 27]. Some cattle networks have, indeed, degree distributions which are almost scale-free [23, 28, 29]. Switzerland’s cattle industry is characterized by high market activity. Even though relatively few in numbers, cattle markets occur at different periodicities and have a wide range of catchment areas. They also vary in terms of veterinary regulations. The markets events range from weekly or biweekly regional trade markets that last a few hours, with a high proportion of animals being taken to slaughter, to yearly national or international events that last a whole weekend.

In addition to cattle markets, the practise of summering herds on high alpine pastures (alps) represents another type of gathering event—much smaller in extent than the markets but also much more numerous. The annual practice of summering to access forage at high pastures dates from ancient times and about half of all Swiss cattle farms participate in it [30]. The animals are walked up on foot in April/May and down in September/October. Usually, the animals from different farms form a single herd that roams during the day and overnights in a single stable with shared milking facilities. Thus, alps can be considered as another type of gathering event where disease could be transferred [31, 32]. In contrast to that of the markets, the periodicity of the alps is yearly, and their duration is around three months. As small gathering events, alps can be expected to contribute to network connectivity, yet due to differences in size and duration their contribution might differ from that of markets. While the impact of markets on network connectivity has been studied previously [21, 23], the role of alpine summering has not yet been evaluated.

While alpine summering has a prominent role in cattle management in Switzerland, Austria, Liechtenstein and the alpine regions of France, Italy and Germany (Bavaria), the practice of bringing herds to higher summer pastures—often leading to a mixing of herds—exist in the Molise, Apulia and Abruzzo regions in Italy, the Pyrenees, the Cantabrian mountains in Spain, the Caucasus and Pontic mountains, the Zagros mountain range in Iran, along the Himalaya and Hindu-Kush mountain ranges, in Norway and Sweden, and—to a lesser extent— also in Wales, Scotland and Ireland. In the case of a severe disease outbreak, or an emergent disease, it would be crucial to know how both types of traditional practices—markets and summering—contribute to disease spread. Here we aim to quantify the relative importance of alpine summering in relation to market related transports for the risk of epidemic outbreaks. We use both a static and a dynamic network analysis approach. Additionally, we investigate seasonal and long-term variation in the network structure of the cattle transport network, its implication for the risk of epidemic outbreaks and its implication for disease surveillance.

## Methods

### (a) Data acquisition

Daily animal transports from January 1st 2012 to December 31st 2015 were extracted from the nationwide Schweizer Tierverkehr Datenbank (Swiss registry for animal movements) using Microsoft SQL Server 2014. For the present analysis, every animal’s history was queried since the start of data recording in 1999 to make sure it was complete. Double entries were excluded. As the slaughterhouses constitute sinks and we are interested in potential disease spread between living animals, the end transports to slaughter were excluded. As we focus on within country transmission, import and export from abroad were excluded.

SQL routines were used to prepare the so-called edge lists containing origin, destination and date of transport for the four years of data. Using information from the Swiss holdings registry [33] holdings were categorized as markets (including trade markets, auctions and exhibitions), dealers, alps and farms. Preliminary data analysis helped to identify one holding originally categorized as farm that showed markedly outlying (10 times higher) values of transports. A closer investigation showed that the respective holder owned three different locations, two with stables and permanent presence of animals and one large hall regularly used for markets but also for other purposes. We, therefore, re-classified the holding at this last location as a market.

### (b) Static network description

We created a static network for each month, where each active holding was represented by a vertex (node) with a directed edge between two nodes representing a movement of one or more animals from the premises of origin to the destination within the respective month. We chose one month to make our results comparable to those from other countries where authors have also analysed monthly networks. It has furthermore been argued elsewhere [23] that the duration of a month will create networks large and dense enough (but not too large and dense) to make a network approach feasible and --at the same time-- it seems unlikely that, given the current surveillance schemes in place, any severe disease could spread undetected for much longer than a month. A holding is considered as ‘active’, when it occurred at least once in the edge list –either as a holding of origin or destination. The constructed network is directed, as origin and destination of the transport are specified, and simple, as multiple movements from one holding to another within a month are not reflected by multiple edges between nodes nor in weights attributed to the edges. The number of animals moved is also not considered, so the links are unweighted, for reasons described in the next section. For each monthly network of the four-year period we calculated the following network measures: density, giant strongly connected component (GSCC), giant weakly connected component (GWCC), clustering coefficient (CC), average path length (APL) and degree assortativity (Assort, see Table 1 for definitions). Furthermore, we calculated for each holding for each month the following nodal network measures: in-degree, out-degree, degree product (in-degree × out-degree), betweenness, in-degree closeness centrality, and out-degree closeness centrality (see Table 1 for definitions).

In order to evaluate the relative importance of the different holding categories, we constructed three further ‘reduced’ networks by consecutively removing markets (and all edges leading from or to markets), dealers (and all edges leading from and to dealers), and alps (and all edges leading from or to alps) from the network. Hence, apart from the full network we got one network without markets, one network without markets and dealers and one network without markets, dealers and alps (farms only).

### (c) Dynamic network measures

Static networks, where edges represent the accumulated movement activity over an extended time do not acknowledge the temporal order of movement events. This can be problematic, specifically when it comes to modelling transmission processes on the network [15–17, 19]. We, therefore, calculated for each node the so-called accessible world [15, 34] or outgoing contact chain [19, 35, 36] over a 30-day period --taking the daily time structure stamps of the events into account--, starting with the first day of each month. The outgoing contact chain is constituted by all destination holdings that can be reached from a certain holding, with the following assumptions: 1) an outbreak would spread undetected and uncontrolled for 30 days, 2) a holding can reach another holding if an animal is transported from that holding to the other holding, 3) at the arrival of an infected animal, all animals at a particular holding get infected immediately and are able to further transmit the infection when leaving the holding, 4) transports are recorded on a daily basis and a holding can only transmit the disease to other holdings, when it was itself infected before animals were moved from it to other holdings, 5) when there were transports from and to a holding on the same day, we assumed that animals were leaving the holding after new animals arrived; i.e. animals arriving transmit the disease to animals leaving on the same day. In this respect, this calculation represents a simple Susceptible-Infectious (SI) model [37, 38] for a worst-case scenario of an epidemic disease that is transmitted via animal movements, only. With these stringent assumptions, the process does not precisely model any specific disease, but it is rather to be seen as a generic model. Importantly, as this process does not incorporate any stochastic element, an exact value can be calculated for each node, given a specific network.

While the outgoing contact chain is a rather generic infection process model, its assumptions are reasonably compatible with a fast spreading disease such as Bluetongue, Foot and Mouth Disease or Lumpy Skin Disease –at least for an initial stage, before effective measures are taken. The length of the outgoing contact chain after 30 days, starting at a specific holding A, gives the number of holdings that would get infected under such a transmission process. We refer to this number as the ‘outbreak size’ of holding A. The choice of 30 days is an arbitrary one, though it is intended to make the results comparable to those built on static monthly measures as they are frequently encountered in the literature. This holding’s property is not constant along time but varies with season and year. Preliminary analysis showed that the distribution for this nodal metric is highly skewed, so neither mean nor median values give a sensible network-wide summary statistic for the overall risk of the outbreak of a large-scale epidemic. Thus, to derive a network-wide measure for outbreak risk, we calculated the proportion of the total number of active nodes with an outbreak size of 100 or larger (which we call hubs thereafter). This measure is conceptually similar to the reachability ratio proposed in [16, 39] and presented in [40], however it differs insofar as these authors present averages and maximum reachability ratios calculated over all nodes. Associations between different measures were assessed by computing Spearman correlation coefficients and generalized linear regression models.

### (d) Stochastic outbreak simulations

To gauge the difference between predictions based on static and dynamic network descriptors, we added a stochastic simulation study that compared detection rate and outbreak size at detection of targeted surveillance based on static and dynamic network measures. As a baseline or reference, we also considered random surveillance. For this simulation disease was seeded on one randomly chosen holding out of all active holdings of the respective year and disease spread was modelled as a stochastic process with the disease spreading from one holding A to another holding B on a given day with probability *p* = 0.8 if a movement of animals from A to B occurred on that day. The number of animals transported from one holding to another was not considered here. Incubation time was assumed to be zero, meaning that on the very day when one or more contagious animals were moved to a holding, animals on that holding can get infected and can also spread the disease further if animals were moved to another holding on the same day or later. Additionally, each day the disease spreads to a randomly chosen holding with probability *q* = 0.05, acknowledging that the disease can also be conveyed by other means than movements of contagious animals. Note that this is a very simple model, not taking into account specific infection dynamics of existing diseases. The parameter values for p and q were arbitrarily chosen, p being a high value and q being a low value. The maximum time for disease spread was set to 30 days with daily updates of infected holdings. Inspired by the work of [41], for each simulation a small number (*n* = 100) of holdings was selected as surveillance targets. Surveillance targets were either chosen randomly from all active holdings (random surveillance), randomly chosen from holdings with high scores for one of the six nodal network metrics (top 5% percentile, static targeted surveillance), or randomly chosen from all holdings with an outbreak size of 100 or more (dynamic targeted surveillance). We use outgoing measures rather than ingoing for convenience. A similar analysis could be carried out using the equivalent ingoing metrics however, as our aim was only to compare the performance of static versus dynamic metrics, this should be of no further concern, here. The simulation was repeated 1000 times for each month of the study period. As measures of the effectiveness of the surveillance scheme we evaluated (i) how often the epidemic was detected at one of the surveillance targets within 30 days, and (ii) the number of infected holdings at the time of detection.

### (e) Software packages and code

Monthly static network measures were calculated using the software R (version 3.1.2) -package igraph (version 3.2.2, https://r-project.org). The plot for the principal component analysis was made using packages ggplot2 and ggfortify. Calculation of the outgoing contact chain and stochastic simulations were done with the software *Mathematica* version 10.2 from Wolfram Research Inc.

## Results

**Fig. 1:**
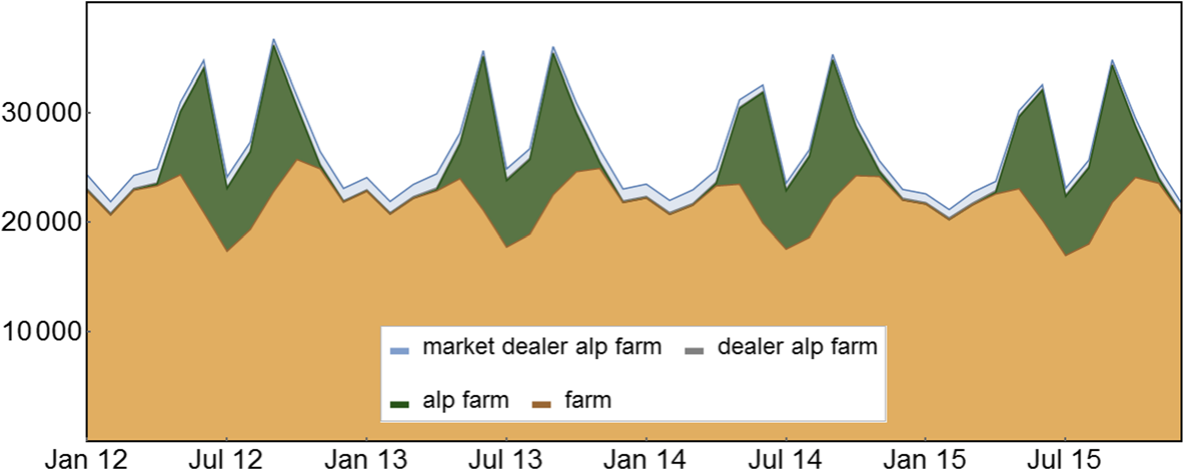
Number of active holdings per month for each of the four scenarios (full network, blue, excluding markets and dealers in green and excluding markets, dealers and alps in brown)

The total number of active holdings per year decreased slightly from 47,212 holdings in 2012 to 44,688 holdings in 2015. Out of those holdings 84.1% were farms, 15.1% alpine pastures, 0.24% dealers and 0.58% markets. The decrease in the number of holdings over the years is mainly due to a loss of active farms at a rate of about 800 farms per year, reflecting the general consolidation trends in the European livestock economy. In total, the Tierverkehr Datenbank recorded between 619,273 transports of animals from one holding to another in 2012 and 598,659 transports in 2015 (Table 2). During the four-year study period 52.4% of Swiss cattle farms participated in the summering practice. Overall 20.2% of the alps hosted animals from a single farm, while the remaining 79.8% of alps hosted animals from several (median: 4 farms, inter quartile range: 2–8) farms. Likewise, 44.2% of cattle farms participated in markets by either selling or purchasing animals at markets. At a typical market, one can find animals from 120 (median; IQR: 44–262) different farms. The number of active holdings (Fig. 1) shows a clear seasonality with one peak in early summer and a second one in autumn, coinciding with the summering pattern. The seasonal pattern mainly disappears after excluding alps except for a trough in summer that is still visible for the farms-only data.

**Table 2:** Average number of animal transports between holding categories per year

	Farm	Alp	Dealer	Market
Farm	415,170	45,690	6066	67,028
Alp	64,808	4342	111	483
Dealer	2730	26	129	100
Market	7070	61	50	22

### (a) Monthly static networks

**Fig. 2:**
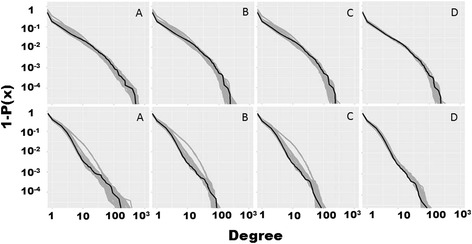
Cumulative probability distributions of unweighted indegree (upper panels) and outdegree (lower panels) for the four networks considered (**A** full network, and successively excluding **B** markets, **C** dealers and **D** alps (farms only))

**Fig. 3:**
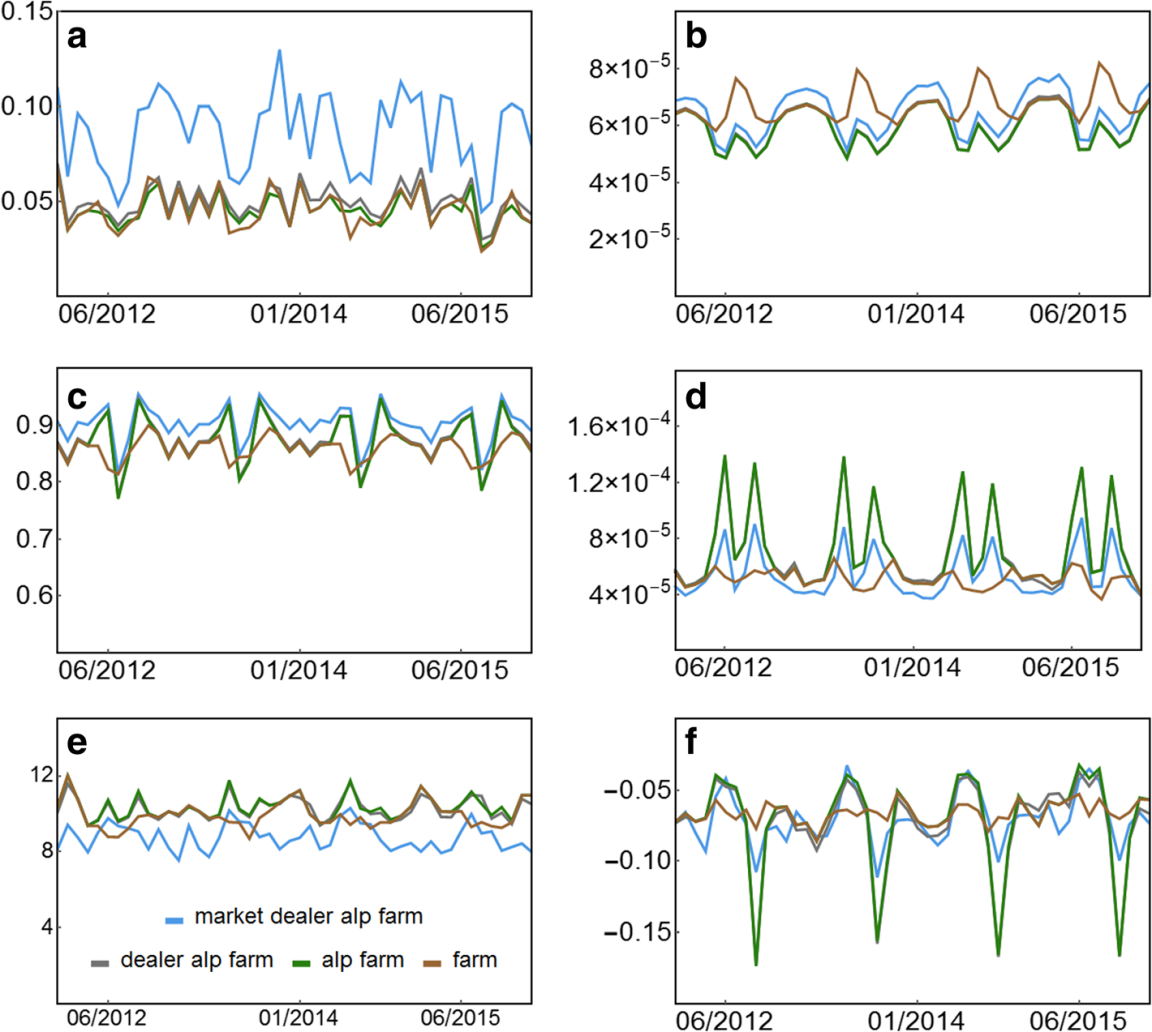
Monthly static network measures, (**a**) giant strongly connected component GSSC, (**b**) density, (**c**) giant weakly connected component GWCC, (**d**) clustering coefficient, (**e**) average path length, (**f**) assortativity, starting from Jan 1st 2012 to December 31st 2015. Complete network in blue, network without markets in grey, network without markets and dealers (alps and farms network) in green and finally without alps (farms only network) in brown. Please note that the grey line is hardly visible as it is under the green line

For each month of the four-year study period we calculated static network measures for the directed transport networks. The monthly degree distributions are highly skewed towards minimum values, (Fig. 2). Deviations from a linear trend in these double-logarithmic plots can be observed. Specifically, the in-degree distributions declined faster than a power-law distribution, similar to the networks described in [42]. With respect to the monthly static network measures (Fig. 3, panels a and c), both GSCC and GWCC vary strongly with season showing a trough during the summer months of July and August when the summering practice keeps animals high up in the pastures. The exclusion of the markets and dealers almost halves the GSCC from 8.64 to 4.54% and reduces the GWCC only slightly from 90.2 to about 85.9%. For both GWCC and GSCC the seasonal pattern largely disappears with the additional exclusion of the alps.

With very low values, the density increases in winter and in the farms-only network in summer (Fig. 3, panel b). The clustering coefficient increases in spring and autumn, especially after having excluded the markets and dealers. The seasonality also disappears with the additional exclusion of the alps (Fig. 3, panel d). Accordingly, the APL increases from a mean of 8.77 for the full network to 9.95 for the farm subnetwork (Fig. 3, panel E). The negative values of the disassortativity index indicate a tendency of high degree nodes to be linked to low degree nodes. The full network (Fig. 3, panel f, blue line) shows a clear seasonal pattern with maximal disassortativity each September. These spikes of highest disassortativity accentuate further when excluding markets and dealers (Fig. 3, panel f, green line), but disappear after exclusion of the alps, suggesting that the markets might render the network overall less disassortative. In any case, the values become closer to zero for the network composed of farms only. In conclusion, the alps are responsible for the marked seasonality in network properties and this trend becomes stronger when excluding markets and dealers. The farms only subnetwork is devoid of a clear seasonal fluctuation except in summer when the density shows maximum values.

**Fig. 4:**
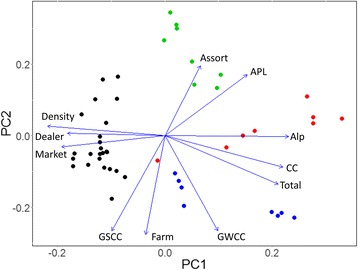
Principal component analysis of network measures and number of holdings for the 48 monthly networks. Black: Winter (November–April), red: Spring (May–June), green: Summer (July – August), blue: Autumn (September–October), Assort: degree assortativity, APL: average path length, CC: clustering coefficient, Dens: density, Tot: total number of active holdings per month; Farm, Alp, Dealer, Market: number of active holdings per month in each category

The seasonality in the network characteristics becomes also apparent in a principal component analysis (PCA) of network-wide measures and numbers of active holdings per category over the 48 months of the study. The first two components explained together 78.9% of the variation (PC1: 50.8%, PC2: 28.1%) and the bi-plot shows that these two components together clearly discern the different seasons of the yearly cycle, with the winter months (November–April) loading low and Spring (May–June) loading high on PC1, while Summer (July – August) loading high and Autumn (September–October) loading low on PC2 (Fig. 4).

### (b) Dynamic network measures

**Fig. 5:**
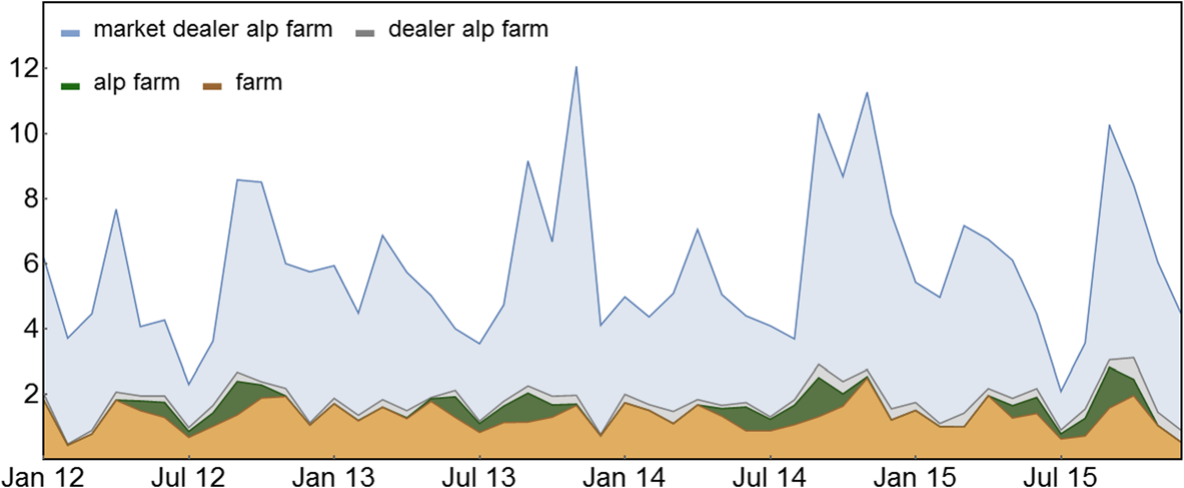
Percentage of total active holdings that render outbreaks larger than 100 other holdings after 30 days of disease spread, calculated at the start of every month from January 1st 2012 to December 31st 2015. Different colors depict the different scenarios, all holdings (upper line), markets contribution (blue area), dealers contribution (grey area), alps contribution (green area), and finally, farms only in brown color

For each holding we evaluated the outgoing contact chain over 30 days, starting at the first day of each month. The length of this chain can be interpreted as a measure for the maximal outbreak size after 30 days considering a highly contagious disease requiring close proximity between individuals for transmission. The outbreak sizes per holding within a single month varied considerably from one, indicating that there was no transmission from this holding within 30 days, to 3754. Outbreak sizes for holdings also varied in the course of the year: 90.7% of all those holdings which had an outbreak size of 100 or larger during one month had an outbreak size of only one during another month of the same year. The distribution of outbreak sizes is highly skewed, with on average 54.5% of the holdings having an outbreak size of one (i.e. there was no transport to any other holding in that respective month) and 85.1% had an outbreak size of less than ten, meaning that even within 30 days of undetected transmission, a highly contagious disease would not have spread to more than ten holdings. We refer to holdings with an outbreak size of 100 or larger as ‘hubs’, as such holdings have the potential to convey a disease to many holdings at any time of an epidemic outbreak. Note, that the categorization of a node of being a hub or not can change from month to month depending on the actual transports and, consequently, the contact chain of the respective month. The percentage of holdings qualifying as hubs is given in Fig. 5. Depending on the month, only between 2.1 and 12.0% of the holdings qualify as hubs, suggesting that in most cases an outbreak starting at a single holding would spread only to a limited number of other holdings within the focal period. Figure 5 shows, further, a strong seasonality, with peaks in spring and autumn and low numbers in summer and around Christmas and New-year.

In our attempt to quantify the contribution of the different holding categories to the overall connectivity of the network and the risk of epidemic outbreaks we consecutively removed all markets and all transports from- and to markets from the network, then all dealers and finally all alps and all transports from and to alps. The number of hubs for these reduced networks is also shown in Fig. 5, and can be interpreted as an estimate for outbreak risk if, for disease-control or other reasons, a certain practise, like the conduct of markets or the summering on alpine pastures, would have been banned or given up. As indicated by the light blue area, removing markets from the network has a substantial impact on the number of hubs, despite making up only a tiny proportion of all holdings. In contrast, dealers, who make up a similar small proportion of the population of holdings, have a much smaller impact (light grey area). Simulated removal of alps from the transport network further reduced the number of hubs during late spring and autumn –at the times when animals were brought to the summer pastures and when they returned to the farms. On the other hand, it had no effect during winter, when the alps were closed and during the summer months, when summering practice seems to lead to an overall reduction of movements and, hence, outbreak risk.

### (c) Associations between different metrics

#### (i) Network-wide measures

A linear regression model with the number of hubs (i.e. holdings with an outbreak size of ≥100) as dependent variable and the monthly network-wide measures GSCC, GWCC, clustering coefficient, average path length and assortativity and their interactions as independent variables could explain over 80% of the total variance (adjusted R^2^ = 0.805, *n* = 48). The best single predictor for the number of hubs was the GSCC with R^2^ = 0.551. The absolute number of movements between holdings and the number of active holdings per month were less reliable predictors for the number of hubs with R^2^ = 0.208, and R^2^ = 0.576 respectively. The number of active farms was the single best predictor: R^2^ = 0.437. Most of the static monthly network measures were correlated with the number of active holdings per month and the number of hubs per month (Table 3).

**Table 3:** Correlation coefficients between the monthly (*n* = 48) number of active holdings (total, number of active farms, alps, dealers and markets) and static monthly network measures (GSCC: Giant strongly connected component, GWCC: giant weakly connected component, CC: clustering coefficient, APL: average path length, Assort: degree assortativity)

	Total	Farm	Alp	Dealer	Market
Total		0.25	0.88	−0.64	−0.67
Farm			−0.23	0.18	0.25
Alp				−0.73	−0.81
Dealer					0.52
GSCC	−0.03	0.76	−0.40	0.29	0.40
GWCC	0.72	0.69	0.39	−0.27	− 0.23
Density	−0.90	− 0.02	−0.89	0.68	0.66
CC	0.94	0.06	0.91	−0.69	− 0.73
APL	0.35	−0.47	0.58	−0.41	−0.44
Assort	−0.01	−0.34	0.16	−0.06	− 0.28

#### (ii) nodal network measures

Given the heterogeneity of the transport network and the variation in outbreak size between individual holdings, we asked to what extent nodal network measures (i.e. measures attributed to individual holdings) can be predictive of the outbreak size for a holding in a given month. A generalized linear model with outbreak size as the dependent variable, a log-link function (family Poisson), month and all six static monthly nodal measures (see below) and their interactions as independent variables resulted in an adjusted R^2^ = 0.199, while post-hoc models with a single network measure, each, delivered even lower proportions of variance explained: in-degree R^2^ = 0.003, out-degree R^2^ = 0.021, degree product R^2^ = 0.003, betweenness centrality R^2^ = 0.008, in-degree closeness R^2^ = 0.038, out-degree closeness: R^2^ = 0.108. All these associations between static nodal measures and outbreak size are far too weak for making them reliable predictors for the outbreak size to be expected for a disease spreading from a specific holding. In conclusion, simple network measures as the monthly number of active holdings as well as monthly static network measures are informative about the overall outbreak risk in a given month. However, nodal measures of monthly networks do not inform about the danger emanating from a specific holding.

### (c) Surveillance examples

In order to gauge whether holding characterization based on dynamic network measures can be of practical importance, we ran a simulation where sentinel holdings were either selected based on the outgoing contact chain, static network measures of monthly networks, or randomly selected from all active holdings. Surveillance based on dynamic measures (randomly selecting 100 holdings from all holdings with outbreak size of 100 or more) detected an epidemic on average in 83.5% of the simulations, while the detection rate was markedly lower for both static targeted surveillance, and random surveillance. Furthermore, the median outbreak size at the time point of detection was lower for the dynamic targeted surveillance than for any other scheme (Table 4). The higher efficiency of surveillance based on the outgoing contact chain is according to our expectations: as outbreak size reflects the accessible world, taking into account the temporal structure of the movements, this measure allows to identify those holdings from which a disease would spread rapidly. Surprisingly, surveillance based on monthly network measures was even less effective than surveillance based on randomly selected sentinel holding. At the moment we do not have an explanation for this finding. This simulation does not constitute a systematic investigation into surveillance strategies, as we employed only a rather simple transmission model and because we confined our investigation to a single set of parameter values instead of exploring the parameter space more broadly. However, these results can at least deliver a proof of concept.

**Table 4:** Comparison of the effectiveness of different surveillance schemes. IQR: interquartile range

Sentinel selection	Detection rate % (median)	IQR	Outbreak size (median)	IQR
outgoing contact chain	83.5	72.4–92.9	50	35–89
in-degree	54.7	32.2–67.2	104	56–173
out-degree	52.6	40.7–66.4	132	49–214
degree prod	56.0	42.4–77.6	114	54–158
betweenness	54.6	31.7–68.7	91	34–159
in-closeness	64.5	38.7–81.2	85	47–151
out-closeness	52.1	35.9–70.3	118	41–208
random	70.3	57.7–85.6	112	57–143

Epidemic outbreaks were simulated using stochastic simulations with 1000 repetitions for each months and each scenario. Surveillance was based on 100 holdings which were either randomly selected from all holdings with an outgoing contact chain larger than 100, from all holdings in the top 5% percentile for one of the static network measures in the respective month, or from all active holdings

## Discussion

We found strong seasonality for several network measures –both static measures for monthly networks and dynamic measures like the proportion of hubs. For example, in autumn the risk of larger outbreaks is five times larger than in summer. Fluctuations in monthly static network measures such as the GSCC are in line with this picture.

The alpine summering practice has a strong impact on the seasonal changes in the transportation network. Simulated removal of alps from the network reduces seasonal variations in the connectivity (GWCC), clustering and assortativity substantially. Yet, when it comes to the risk of epidemic outbreaks, markets play a much more prominent role than the alps. This has an important implication with respect to outbreak control interventions, such as transport bans.

By using unweighted data, our focus is on fast spreading and highly contagious diseases, where the transport of a single contagious animal would be sufficient to infect all other animals in the holding of destination. Disease specific models that take the transmission dynamics and etiopathology of the specific disease into account would be required to evaluate the contribution of holding types in the case of slow spreading diseases [18, 43]. Furthermore, we would like to mention that our approach (simulated removal of nodes, see also [12, 29, 44, 45] for further use of this approach) does not account for any potential increased activity of the holdings that remain in the network when excluding a particular holding category. This is, of course, a simplification and a study by Robinson and colleagues [46] reported that after the introduction of mandatory standstill periods in Great Britain (a response to the foot and mouth disease epidemic in 2001), movement patterns have shifted constantly resulting even in an increase of the GSCC and hence the potential size of the next epidemic outbreak.

The prominence of markets as potential hubs for disease transmission, as it is suggested by their contribution to the overall outbreak sizes, is well in line with results found in other studies [22, 23]. It is important to keep in mind that large market events are subject to strict regulations to prevent disease spread, including medical surveillance and spatial structuring to minimize contact between animals from different holdings. Thus our findings should be interpreted as potential risk, if no control measures and regulations were in place, but not as a measure for the actual risk given current best practice. In this respect, our results clearly stress the importance of the precautionary measures taken at large markets and auctions. Network-wide metrics of monthly transport networks were clearly correlated with the proportion of hubs (i.e. outgoing contact chains with a length of 100 or more), which we took as a risk measure. However, the number of active holdings, specifically markets and alps, also showed a strong seasonal pattern that was correlated with outbreak risk. From a mechanistic perspective, it is clear that changes in the risk of epidemic outbreaks are a consequence of changes in connectivity of the network, which is a consequence of the seasonal cycle. Thus, while several network measures allow predictions about times of increased outbreak risk, the same feat could be achieved by simply counting holdings per category, or –even more simply— consulting the calendar. Nodal static network measures of the monthly transport networks give only poor predictions for the risk emanating from a specific holding. Of the six static nodal measures considered, only out-degree closeness had a noteworthy predictive value for outbreak risk.

The reason for this discrepancy can be found in the temporal dynamics of the network. If a transport from holding B to holding C is recorded for time point *t*, and another transport from holding A to holding B for the time point *t* + 1, then holding A is connected to both holdings B and C in the monthly static network. However, due to the temporal order of the connections a disease could not spread from A to C. The outgoing contact chain takes this temporal dynamic into account and, as such, it gives a more sensible network metric reflecting its potential contribution to an epidemic outbreak. We simulated outbreaks with a simple model, which contained two stochastic components: one for the transmission of the disease due to animal transports from one holding to the other, and a second one for transmission due to environmental contamination or other unknown reasons. Yet, targeted surveillance based on the dynamic outgoing contact chain could detect epidemics more often and more effectively than both targeted surveillance based on holdings selected based on static network properties and random surveillance. Our results are in line with recent propositions for selection criteria of surveillance targets based on cluster analysis of the transport network [18, 41]. Even though the methodologies differ in details, all these studies accumulate evidence of how promising dynamic or temporal approaches are for risk evaluation and surveillance.

## Conclusions

We provided a detailed description of the Swiss cattle transport network. Static descriptors of monthly transport networks give only poor predictors for the outbreak risk emanating from individual holdings; yet a dynamic measure based on time-stamped data—the outgoing contact chain—can give better risk estimates and could help to improve surveillance schemes. Using this measure we find evidence that even in a country with intense summering practice, markets continue being the prime risk factor for the spread of contagious diseases.

## Abbreviations

APL: Average path length
Assort: Degree assortativity
CC: Clustering coefficient
GSCC: Giant strongly connected component
GWCC: Giant weakly connected component
IQR: Interquantile range
